# TNFα in MS and Its Animal Models: Implications for Chronic Pain in the Disease

**DOI:** 10.3389/fneur.2021.780876

**Published:** 2021-12-06

**Authors:** Aislinn D. Maguire, John R. Bethea, Bradley J. Kerr

**Affiliations:** ^1^Neuroscience and Mental Health Institute, University of Alberta, Edmonton, AB, Canada; ^2^Drexel University, Philadelphia, PA, United States; ^3^Department of Pharmacology, University of Alberta, Edmonton, AB, Canada; ^4^Department of Anesthesiology and Pain Medicine, University of Alberta, Edmonton, AB, Canada

**Keywords:** pain, cytokine, TNF-α, EAE (experimental autoimmune encephalomyelitis), NFkapapB, MAP kinase (MAPK), autoimmune disease

## Abstract

Multiple Sclerosis (MS) is a debilitating autoimmune disease often accompanied by severe chronic pain. The most common type of pain in MS, called neuropathic pain, arises from disease processes affecting the peripheral and central nervous systems. It is incredibly difficult to study these processes in patients, so animal models such as experimental autoimmune encephalomyelitis (EAE) mice are used to dissect the complex mechanisms of neuropathic pain in MS. The pleiotropic cytokine tumor necrosis factor α (TNFα) is a critical factor mediating neuropathic pain identified by these animal studies. The TNF signaling pathway is complex, and can lead to cell death, inflammation, or survival. In complex diseases such as MS, signaling through the TNFR1 receptor tends to be pro-inflammation and death, whereas signaling through the TNFR2 receptor is pro-homeostatic. However, most TNFα-targeted therapies indiscriminately block both arms of the pathway, and thus are not therapeutic in MS. This review explores pain in MS, inflammatory TNF signaling, the link between the two, and how it could be exploited to develop more effective TNFα-targeting pain therapies.

## MS and Its Mouse Models

### MS Background

Multiple Sclerosis (MS) is an autoimmune disease characterized by aberrant immune cell activity leading to inflammation and demyelinating lesions of central nervous system (CNS) (1–3). It's causes, while not fully understood, are likely a combination of genetic and environmental factors (4). The MS disease course can follow multiple trajectories. Primary progressive disease worsens steadily from onset. Progressive relapsing disease increasingly worsens but with some relapsing and remitting characteristics, meaning there are periods where symptoms worsen, then improve again. Most patients have a biphasic disease course, wherein they initially present with a relapsing-remitting phenotype, but as the disease progresses there is a switch to the secondary progressive phenotype and disability continually worsens (2, 5). There are numerous symptoms and comorbidities associated with MS, which can affect sensory, motor, and cognitive modalities. One of the most debilitating ailments experienced by MS patients is chronic pain (6, 7).

### Pain in MS

Pain is a common feature for many patients diagnosed with MS (6, 8). The pain MS patients may encounter includes chronic headache, sudden neck pain called Lhermitte's sign, trigeminal neuralgia, extremity pain and hypersensitivity due to neuropathy (central or peripheral) (9). Pain is one of the most devastating comorbidities of MS, significantly interfering with daily life and yet, there are few treatments available (9–11). This is likely because neuropathic pain (NP), chronic pain that is caused by injury or disease of the nervous system, underlies most pain in MS (12). Neuropathic pain cannot be treated with typical painkillers such as opioids or anti-inflammatory drugs but instead, is often treated with more non-specific drugs like anti-depressants or anti-convulsants which have severe side effects (9, 11, 13, 14). Treating neuropathic pain in autoimmune diseases is further complicated by a process called sensitization, which occurs in both the peripheral and central nervous system (PNS and CNS). Sensitization can involve both intra and inter-cellular changes that increase pain sensations and allow for the maintenance of pain regardless of disease progression or treatment (15). Studies in similar autoimmune diseases (namely Rheumatoid Arthritis) have demonstrated that pain is often not resolved by disease-modifying treatment, and must be studied and treated separately (16). To study the mechanisms of pain in MS, researchers have turned to animal models which exhibit comparable disease phenotypes in the PNS and CNS, and most importantly, pain.

### Mouse Models of MS

There are several paradigms used to induce MS-like symptoms in laboratory animals. Although MS models have been developed in rats, non-human primates, and even zebrafish, the greatest diversity exists in mouse models (17). MS models are categorized into three main groups, though viral infection, self-antigens that become recognized by the immune system, or toxins that cause demyelination. Theiler's murine encephalomyelitis virus (TMEV) induced disease is the best example of a viral induced model of MS, while experimental autoimmune encephalomyelitis (EAE) represents the prototypical antigen induced disease model, and demyelination is induced by cuprizone or lysophosphatidyl choline (LPC) administration (Table 1).

**Table 1:** Mouse models of Multiple Sclerosis.

**Name**	**Induction method**	**Disease phenotype**	**Does it cause Pain?**	**References**
TMEV	Viral infection	Biphasic	Yes	(18–20)
SJL/J EAE	Immunization with PLP_139−151_	Relapsing-remitting	Yes	(21, 22)
Transgenic EAE	Mice with genetically manipulated T and/or B cells	Primary progressive or relapsing-remitting	Yes	(23–26)
Pertussis/CFA EAE	Immunization with MOG emulsified in CFA, then pertussis	Primary progressive or relapsing-remitting	Yes	(27, 28)
Pertussis/QuilA EAE	Immunization with MOG emulsified in QuilA, then pertussis	Relapsing-remitting	Yes	(29–33)
Non-pertussis EAE	Immunization with MOG in CFA, no pertussis	Primary progressive	Yes	(34)
Cuprizone	Administered in diet for 5+ weeks	Demyelinating	Yes	(35)
LPC	Peripheral or central injection	Demyelinating	Yes	(36–38)

Due to its induction method, mice infected with TMEV develop a biphasic disease phenotype that is useful for studying the viral contribution to MS (18, 19). EAE on the other hand, refers to a variety of ways to induce immune activation and demyelination that mimic MS pathophysiology. This is most often achieved by immunization with a myelin antigen. For example, EAE induced in Swiss Jim Lambert (SJL) mice with a fragment of proteolipid protein (PLP_139−151_) causes a relapsing-remitting disease phenotype (21). A primary progressive phenotype can be induced by immunization with myelin oligodendrocyte glycoprotein (MOG_35−55_) emulsified in an adjuvant such as CFA to trigger an immune response to myelin (27, 28, 39, 40). By modifying the concentration of MOG_35−55_ and the adjuvants used to induce EAE, a relapsing-remitting phenotype in C57Bl/6 mice can also be generated (29, 30, 41). MOG EAE immunization protocols are normally followed by injections with pertussis toxin to facilitate blood brain barrier breakdown (39), but this step can also be omitted (34). QuilA can also be used in place of Complete Freund's Adjuvant (CFA), the most used adjuvant in EAE models, to generate a relapsing-remitting phenotype (31).

Transgenic EAE is yet another method of mimicking MS in mice. T and/or B cells in these mice are genetically manipulated to react to MOG, and different strains have been developed to produce either a primary progressive or relapsing-remitting phenotype (23–26). Lastly, demyelination can be caused by either consumption of the copper chelator cuprizone which preferentially causes oligodendrocyte cell death (42, 43), or injection of LPC which integrates into membranes and disrupts myelin (44, 45). These models are useful to study demyelination separately from other MS disease processes. Although there are many ways to induce MS-like symptoms in mice, and each have their own strengths and weaknesses in modeling CNS lesions, demyelination, axonal damage, immune cell activation, they all produce pain (20, 22, 35).

### Pain in MS Models

Animal models of MS have enabled researchers to study the mechanisms of chronic pain associated with the disease as the animals develop similar pain phenotypes to people with MS (46). Like MS patients, mice with EAE also exhibit cold and mechanical hypersensitivity, trigeminal neuralgia, and even sex differences in pain (9, 47, 48). Animals with TMEV and EAE exhibit hypersensitivity to painful and non-painful stimuli called, called hyperalgesia and allodynia, respectively (20, 46, 49). Interestingly, TMEV animals present with sex differences in pain, with females developing hypersensitivity more quickly than males (20). This sex difference is important as it allows researchers to better understand sex differences in human MS. In a foundational study of pain in EAE, animals exhibited heat-induced hyperalgesia not only when the disease was induced by immunization with a myelin peptide emulsified in CFA, but also when T cells from EAE mice were transferred to naïve mice (50). The cuprizone model has historically been studied less in the context of pain, but a recent study using electrical stimulation-induced paw withdrawal suggests there is a pain phenotype in cuprizone mice (35). LPC injection has also been associated with pain, but more commonly in the context of nerve or spinal cord injury (36–38).

One mechanism that may be responsible for some aspects of pain in MS animal models is immune cell activation and cytokine release, generating peripheral and central sensitization. Tumor necrosis factor alpha (TNFα) is of the most prominent pro-inflammatory cytokines present in MS and EAE, and it also has strong associations with many other chronic pain conditions (51–53).

## TNFα Structure and Function

### TNFα Signaling

**Figure 1:**
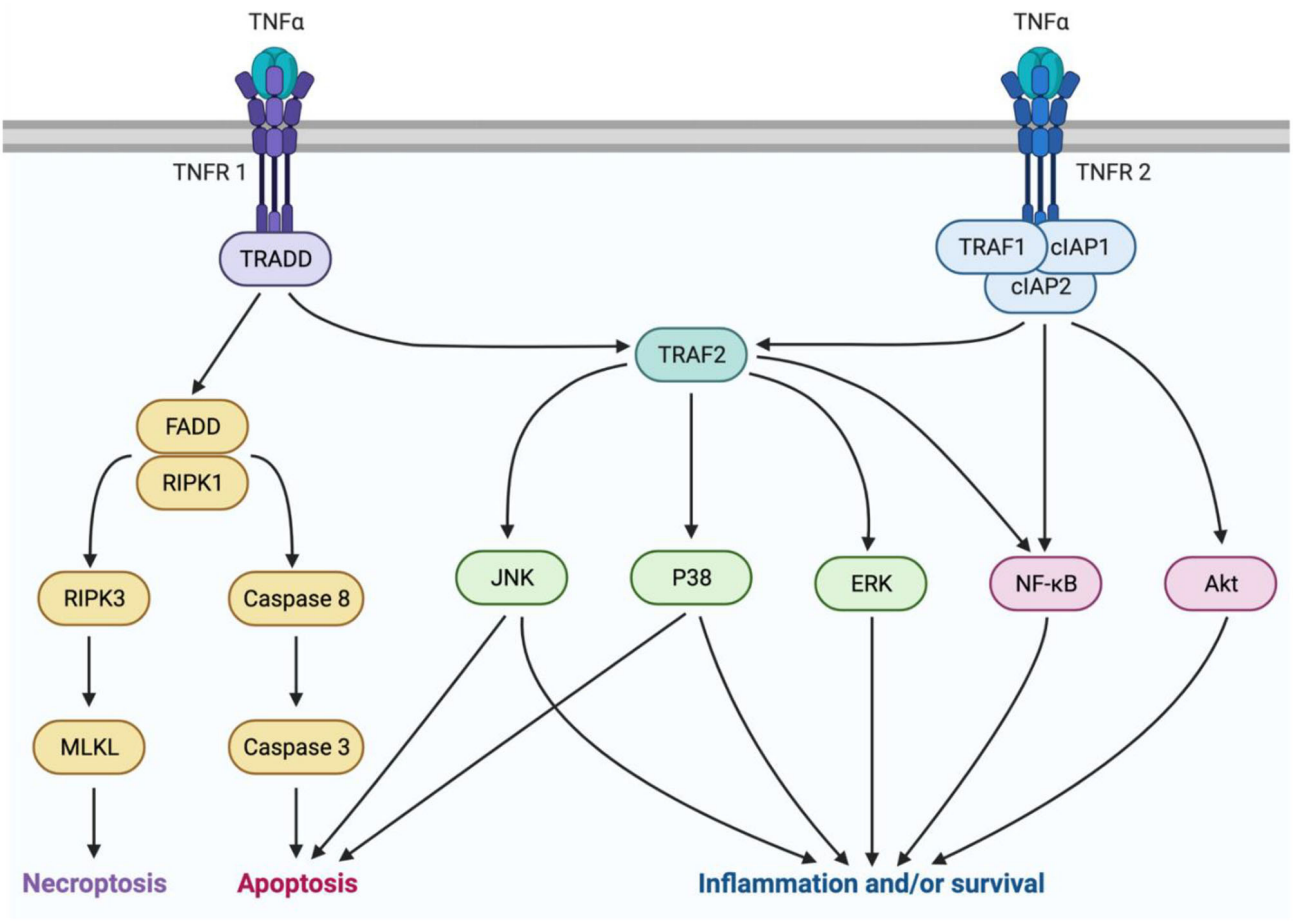
TNFα signaling is mediated by two isoforms of the cytokine and two receptor subtypes. sTNFα can interact with TNFR1 to mediate either pro-inflammatory or pro-death signaling. Pro-death signaling occurs through recruitment of FADD and RIPK1, which can either lead to necroptosis trough RIPK3 and MLKL activation, or apoptosis through caspase8 and caspase 3 activation. Pro-inflammatory signaling occurs through TRAF2 activation of P38, JNK, or ERK MAPKs, or NFκB. Alternatively, tmTNFα can interact with TNFR2 to mediate pro-homeostatic or pro-inflammatory signaling through TRAF1, cIAPs 1 and 2, and TRAF2. This pathway can be mediated by activation of MAPKs, NFκB, or Akt. This figure was made using BioRender.

TNFα is a pleiotropic cytokine with a multifaceted signaling pathway which can lead to cell death *via* either apoptosis or necrosis, or conversely, to survival and inflammation (Figure 1). TNFα originates in its transmembrane form (tmTNFα), produced by immune cells such as macrophages, monocytes, and lymphocytes (54, 55). Then it may be cleaved by TNFα converting enzyme (TACE) and released into its soluble form (sTNFα) (55). As the main determinant of TNFα isoform availability, TACE overactivity has been linked to inflammatory diseases (56). However, it is not currently a viable treatment target due to its similarity to other matrix metalloproteinases (57). There are two main subtypes of TNF receptors, TNFR1 and TNFR2. TNFR1 is expressed on most cell types and primarily mediates pro-inflammatory and pro-death signaling (58). It can bind both sTNFα and tmTNFα, but is preferentially activated by sTNFα (59, 60). TNFR2 is expressed mostly on immune cells and only associates with tmTNFα (61, 62). This receptor lacks a death domain and is associated with pro-survival and pro-homeostatic signaling (62). Interestingly, during this interaction, tmTNFα also transmits signals back into its host cell (55).

sTNFα molecules act in a trimer and associate with three TNFR1 receptor subunits to activate the signaling complex by endocytosis into the cytoplasm (63). Next, Tumor necrosis factor receptor type 1-associated death domain protein (TRADD) associates with the receptor-ligand trimer. Further complex recruitment by TRADD then determines whether death or inflammation and survival will occur (63). Death signaling ensues if TRADD recruits fas-associated protein with death domain (FADD), and receptor-interacting serine/threonine-protein kinase (RIPK) 1 (64). Cell death occurs by apoptosis if initiator caspases 8 or 10 are recruited, or by necroptosis if RIPK3 and mixed lineage kinase domain-like pseudokinase (MLKL) are recruited (64). The pro-survival factor, TNF receptor-associated factor 2 (TRAF2) can prevent cell death by acting as an E3 ubiquitin ligase to target RIPK1 for degradation (65, 66). TRAF2 then initiates activation of the mitogen-activated protein kinases (MAPKs) P38, c-Jun-N-terminal kinase (JNK), and extracellular signal-regulate kinase (ERK), or the transcription factor nuclear factor kappa B (NFκB) (65). Pro-inflammatory signaling by these factors is a beneficial response to insults such as infection, but it can also be maladaptive, leading to pain (67, 68).

TNFR2 signaling also occurs in a trimeric fashion but rather interacts with tmTNFα, then recruits TRAF2 upon complex endocytosis. In addition, TNFR2 recruits TRAF1 and cellular inhibitors of apoptosis (cIAP1/2). This complex activates pro-survival signals through phosphatidylinositol 3-kinase (PI3K) and protein kinase B (Akt), and activates NFκB and JNK (69, 70). Although TNFR2 lacks a death domain, prolonged JNK activation by TNFR2 can lead to intrinsic apoptosis (65). Despite this ability to cause cell death, TNFR2 signaling is primarily pro-homeostatic and promotes many pro-survival activities including, cell proliferation, migration, and adhesion (71, 72).

### TNFα and Pain

**Figure 2:**
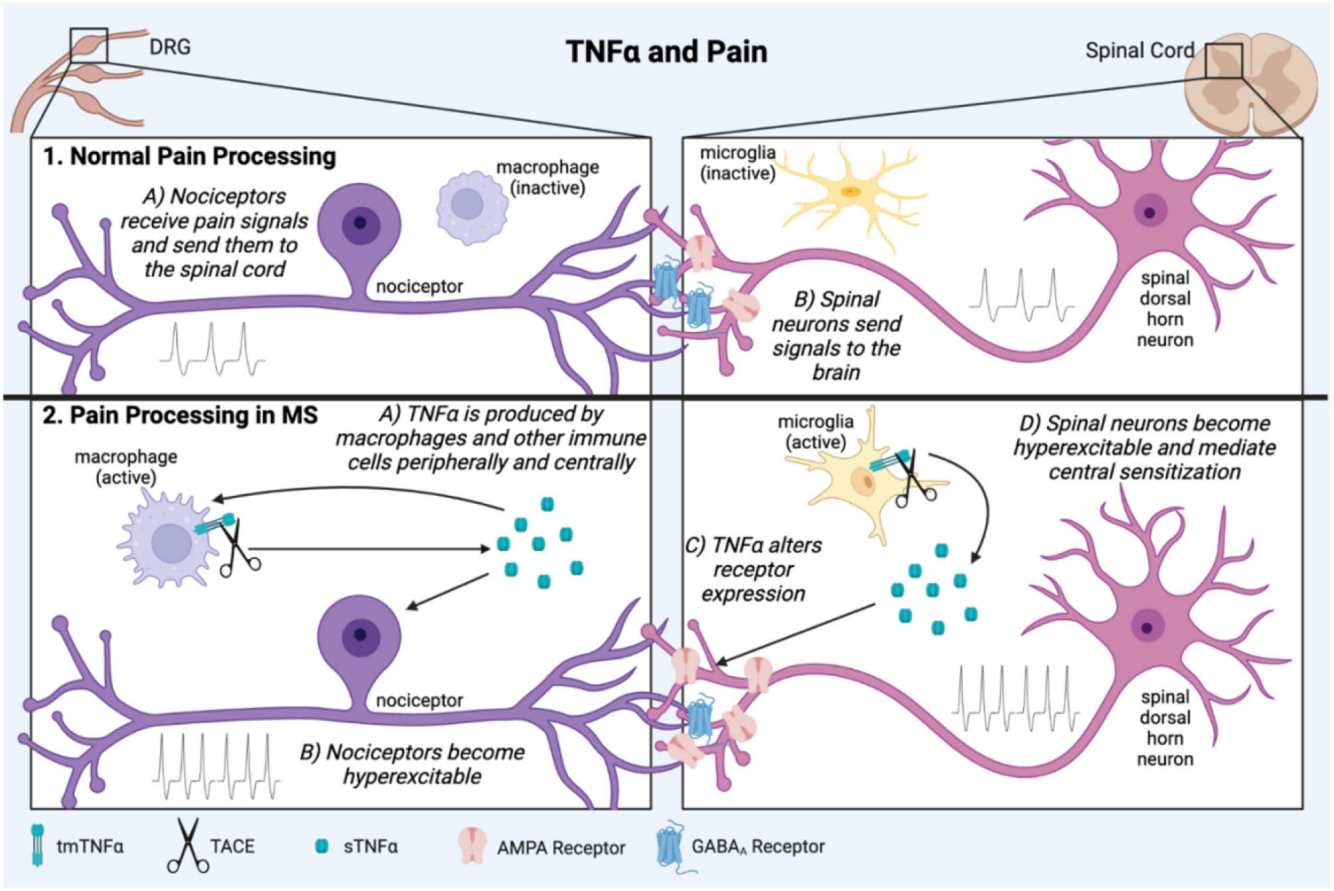
TNFα is involved in pathological pain processing in MS. In normal physiological pain processing, pain signals are sent from the periphery to the DRG, which relays information to the spinal cord then brain (1A,B). In neuropathic pain in MS, inflammation activates immune cells which secrete the pro-inflammatory cytokine, TNFα both peripherally and centrally (2A). It can then act back on the cells which produced it creating a positive feedback loop. TNFα contributes to sensitization of peripheral nociceptors and spinal dorsal horn neurons through mechanisms such as altered excitatory and inhibitory receptor expression (2B,C). These conditions lead to long term central pain sensitization of the brain and spinal cord (2D). This figure was made using BioRender.

TNFα is involved in both central and peripheral mechanisms of chronic pain (73–75) (Figure 2). This has been demonstrated on a pre-clinical level in animal experiments which show that TNFα administration alone is sufficient to cause pain (76–78), and exogenous TNFα administered in animal models of inflammatory pain such as spinal nerve ligation (SNL) can exacerbate pain intensity and duration (79). In more complex animal models of neuropathic pain such as peripheral nerve injury (PNI), TNFα is elevated both centrally and peripherally, and TNF antagonists can be effective in relieving pain (80–84).

Various mechanisms for how TNFα causes pain have been investigated. TNFα produced in response to inflammation can increase excitatory synapse strength and decrease inhibitory synapse strength by altering AMPA and GABA_A_ receptor surface expression on neurons (85). In the hippocampus this hyperexcitation leads to excitotoxicity and neuronal death (85, 86), but in the dorsal horn of the spinal cord it can cause either excitotoxic cell death or sensory sensitization and pain (87, 88). TNFα is also a well-characterized activator of microglia, leading to further secretion of inflammatory mediators (89, 90). This process has been implicated in spinal mechanisms of neuropathic pain (84, 91).

Whether TNFα leads to inflammation or cell death depends on its downstream signaling. For example, in male rats, SNL injury increased TNFα and P38 MAPK expression in the DRG and spinal cord, and inhibition of either TNFα or P38 was sufficient to reduce mechanical allodynia (92). While P38 and JNK MAPK signaling can be pro-inflammatory and pro-survival, they can also lead to intrinsic apoptosis through mitochondria (93). TNFα can also be involved in pain in a secondary manner. In an animal model of intervertebral disc degeneration, TNFα signaling contributed to disc degeneration by inducing apoptosis through caspase 3, and that subsequent disc degeneration caused pain (94).

The detrimental effects of TNF signaling in pain conditions are mediated primarily by TNFR1. Several anti-TNFR1 antibodies have been developed, as have inhibitors of sTNFα used to block TNFR1 signaling. An example of an sTNFα blocker, XPro1595, inhibited hyperalgesia in a CFA model of inflammatory pain (95) and in EAE (96). Additionally, after spinal cord injury, XPro1595 treatment increased TNFR2 expression (97). TNFR2 is considered pro-homeostatic, as evidenced by studies in which TNFR2 agonism has relieved pain after PNI (58, 98). Current evidence thus indicates that sTNFα signaling through TNFR1 is pathological in pain conditions, whereas tmTMFα signaling through TNFR2 is protective.

While animal models have the benefits of being well controlled for age, sex, environment, and the nature of illness or injury, the inherent variability in human populations complicates the study of pain and its treatment. However, TNFα is elevated is a number of painful conditions in humans including chemotherapy-induced neuropathic pain (CIPN) and rheumatoid arthritis (RA) (51). Non-specific TNFα antagonists have shown some effectiveness in relieving pain in RA, but not in all inflammatory pain conditions in which the cytokine might be involved, such as MS (99, 100).

### TNFα in MS and EAE

**Figure 3:**
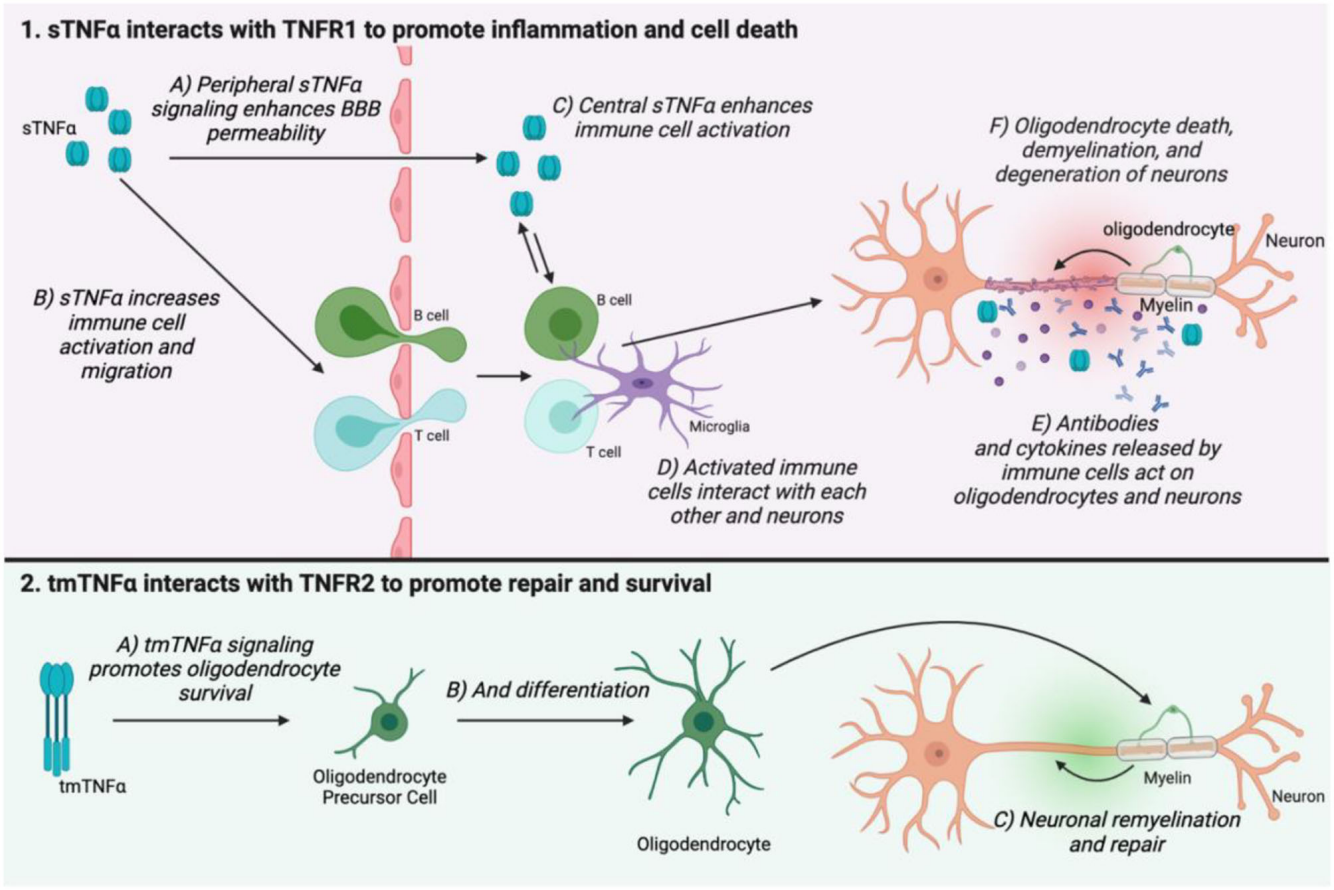
The two TNFα isoforms and their receptors have opposing actions on MS disease progression. sTNFα interacts with TNFR1 in the peripheral nervous system to enhance BBB permeability (1A), as well as increase immune cell activation and migration (1B). TNFα can migrate across the weakened BBB, but it is also produced in the central nervous system where it again promotes immune cell activation (1C,D). Immune cells secrete antibodies and cytokines which mediate oligodendrocyte death, demyelination, and neuronal damage (1E,F). tmTNFα interacts with TNFR2 to promote oligodendrocyte survival and differentiation. This helps to promote remyelination and repair (2C). This figure was made using BioRender.

TNF signaling may be involved in MS pathogenesis through several points of action. TNFα is elevated centrally in MS patients, and this is correlated with disease severity (101). However, it is incredibly difficult to study the precise mechanisms underlying TNF signaling in MS in humans. Much of the proposed actions of TNFα in MS have been discovered through study of animal models such as EAE. In both MS and its animal counterparts, the major pathological landmarks are immune cell infiltration into the CNS, and the development of demyelinating lesions which eventually lead to neuronal death. In EAE, TNFα transport is upregulated at the blood-brain barrier (BBB), as is TNFα expression by mast cells, which are involved in BBB breakdown (102). TNFα also promotes activation of T cells (103, 104), and is upregulated in demyelinating lesions in EAE where it is hypothesized to promote neuronal excitotoxicity and oligodendrocyte death (103, 105). Therefore, TNFα appears to be involved in immune cell activation and infiltration into the CNS, as well as demyelination and axonal injury. The receptor subtype employed in TNF signaling also shapes disease progression in MS and EAE. TNFR1 expression is correlated with disease progression, whereas TNFR2 promotes repair and remyelination through oligodendrocyte survival and differentiation (106–108) (Figure 3).

While central TNF signaling is involved in disease progression in MS and EAE, peripheral TNFα elevation is a likely culprit for the development of chronic pain (52). Infiltration of TNFα-producing immune cells is evident peripherally in EAE (109). Macrophages, for example, both produce TNFα and are strongly affected by it (110). In a pro-inflammatory environment where sTNFα is the dominant isoform, TNFR1 signaling in macrophages can enhance their activation, resulting in a positive feedback loop (111). Primary pain sensing neurons, called nociceptors, express TNFR1 (112), and signaling through this receptor can cause nociceptors to become hyperexcitable, meaning they may be more likely to fire action potentials, and do so more intensely in response to painful stimuli (113, 114). These hyperexcitable nociceptors then signal to the spinal cord and brain, leading to central sensitization, which maintains pain chronically (115). This mechanism for pain has been proposed in other painful conditions like PNI and RA, where treating the peripheral causes of pain is ineffective once central sensitization has been established (115–117). It may also explain why in EAE, pain sensitivity to stimuli occurs early in the disease course, before full disease onset (118). While pain mechanisms in EAE have not been fully elucidated, understanding this model of peripheral and central sensitization will help inform future areas of study and potential treatments.

## Targeting TNFα as an Analgesic Strategy

### Anti-TNF Therapies

There are five non-specific TNFα inhibitors currently in clinical use (Table 2). Infliximab, Adalimimab, Golimumab, and Certolizumab are antibodies that target TNFα, and Etanercept is a soluble recombinant TNF receptor (119, 120). All of these drugs work by binding to and sequestering both the soluble and transmembrane forms of TNFα so they cannot interact with their receptors and initiate signaling cascades (55). Anti-TNF drugs can be beneficial in various types of arthritis, ankylosing sponditis, plaque psoriasis, Crohn's disease, and ulcerative colitis (121). However, there are severe side effects associated with TNF inhibition. TNFα is an important immune-mediator, and blocking its actions can be immunosuppressive, thereby increasing the risk of new infections as well as re-activation of dormant infections (122, 123). There is also evidence these drugs cause demyelination and liver damage (124, 125). The severe side effects of TNF inhibition may be due to blockade of the homeostatic functions TNFα, particularly through TNFR2 signaling. This indiscriminate blockade of TNFα may also help to explain why pain management is lacking with anti-TNF treatment (126–128).

**Table 2:** TNFα-targeted therapies.

**Drug**	**Clinical approval?**	**Type**	**Target**	**Could treat MS pain?**	**References**
Infliximab	Yes	Monoclonal TNFα antibody	Soluble and transmembrane TNFα	No	(124, 129, 130)
Adalimumab	Yes	Monoclonal TNFα antibody	Soluble and transmembrane TNFα	No	(124, 131–133)
Golimumab	Yes	Monoclonal TNFα antibody	Soluble and transmembrane TNFα	No	(124, 134–136)
Certolizumab	Yes	PEGylated antigen-binding fragment TNFα antibody	Soluble and transmembrane TNFα	No	(124, 137, 138)
Etanercept	Yes	Soluble TNFα receptor	Soluble and transmembrane TNFα	No	(124, 139, 140)
Xpro1595	No	Protein biologic	Soluble TNFα	Yes	(96, 97)
R2agoTNF	No	TNFα mutant	TNFR2 (agonist)	Yes	(141, 142)
Nabiximols	Yes	Cannabinoid	Soluble TNFα	Yes	(143, 144)

Anti-TNF therapies have mixed effectiveness in treating pain depending on the condition for which they are used. In a rat chronic constriction injury model of PNI, TNF inhibition reduced mechanical and thermal pain (99, 145). Pain reduction by anti-TNF drugs in PNI has been suggested to occur through an alteration of TNF receptor expression in the spinal cord (146). Anti-TNF treatment with either etanercept or infliximab decreased the TNFR1/TNFR2 ratio, and this correlated with better recovery (145). This finding conforms with the view of TNF receptors which considers TNFR1 to be involved in pathology/damage, and TNFR2 to be involved in repair and homeostasis (147, 148). Although it is unclear how exactly these anti-TNF drugs modulate receptor expression, these findings provide a strong rationale to further investigate TNF receptor modulation in neuropathic pain treatment, and there are currently drugs in development for this purpose (Table 2).

In another peripherally-driven chronic pain disorder, diabetic peripheral neuropathy (DPN), TNF inhibition blocked mechanical but not thermal pain in a rat model (99, 149). Anti-TNF treatment in this model has also improved signs of nerve degeneration associated with advancing DPN, restoring conduction velocity, myelination, myelin basic protein expression, as well as lamellar and axonal organization (150). Based on the findings in these two models, TNF inhibition can affect sensory neuron inflammation and degeneration, as well as spinal TNF receptor expression. It will be important for future studies in PNI and DPN models to address both peripheral and central mechanisms. This may provide a better understanding of how targeting TNF can prevent and/or relieve neuropathic pain.

### Anti-TNF in MS: The Double-Edged Sword

Anti-TNF therapies are generally not only ineffective in treating MS, but they can also worsen disease severity. They are also known to increase the risk of developing MS in patients receiving anti-TNF treatment for other conditions (124, 151, 152). Studies using EAE have provided insight into why universal blockade of TNF signaling can be detrimental in the disease. Anti-TNF molecules sequester both soluble and transmembrane TNFα and block signaling through both TNFR1 and TNFR2 (55). In EAE, inhibition of soluble TNFα signaling through TNFR1 promotes remyelination and axon survival (96). However, transmembrane TNFα signaling through TNFR2 may be neuroprotective (153). TNFR2 signaling supports regulatory T cells (Tregs) (98, 154), and promotes remyelination through oligodendrocyte differentiation (106). Ultimately, while blocking all TNF signaling in MS and EAE can reduce its detrimental effects, it also reduces the beneficial aspects of TNFR2 signaling, leading to a net negative result for anti-TNF therapies in MS and EAE.

Drugs that are more specifically targeted to cell type, TNFα isoform, and TNF receptor type will be necessary to further explore TNF therapies for disease modification and pain treatment in MS (Table 2). For example, in EAE, treatment with a selective TNFR2 agonist reduced motor symptom and pain severity and improved various other hallmarks of the disease (155). Cannabinoids are another potential treatment for MS pain, as they are linked to a preferential reduction sTNFα production by both peripheral and central immune cells (156–158), and have shown promising analgesia in clinical trials (159, 160). Further investigation into similar receptor and/or isoform targeted drugs may make TNF therapy a viable option in MS pain treatment.

## Gaps in Knowledge and Future Directions

TNFα mediates the development of neuropathic pain in many conditions. While indiscriminate TNF inhibition is effective in some human pain conditions and animal models, it is ineffective and can have deleterious consequences in MS. To develop therapies that effectively target TNF signaling to treat pain in MS we must first focus on developing a better understanding of the cell types, receptors, and downstream pathways involved both peripherally and centrally. Research in the EAE model has already led to the development of promising TNFR1 antagonists and TNFR2 agonists. Other components of the TNF pathway such MAPKs and NFκB may also provide points of intervention. These targeted therapies are the future of pain management in MS and other neuropathic pain conditions.

## Author Contributions

AM, BK, and JB conceptualized the review. AM wrote the manuscript. BK and JB edited the manuscript. All authors contributed to the article and approved the submitted version.

## Funding

This work was supported by a Project Grant from the Canadian Institutes of Health Research (FRN-162434) and a Discovery Grant from the MS Society of Canada (EGID-3761).

## Conflict of Interest

The authors declare that the research was conducted in the absence of any commercial or financial relationships that could be construed as a potential conflict of interest.

## Publisher's Note

All claims expressed in this article are solely those of the authors and do not necessarily represent those of their affiliated organizations, or those of the publisher, the editors and the reviewers. Any product that may be evaluated in this article, or claim that may be made by its manufacturer, is not guaranteed or endorsed by the publisher.
